# Functional and phenotypic analysis of CD4 T cell dynamics in peripheral blood of human visceral leishmaniasis patients confers increased frequencies of CD25 expressing regulatory T cells that contribute to disease pathogenesis

**DOI:** 10.3389/fimmu.2025.1676937

**Published:** 2025-11-27

**Authors:** Mohd Kamran, Smriti Ghosh, Pradyot Bhattacharya, Sneha Ghosh Chaudhury, Anirban Bhattacharyya, George Banik, Mohammad Asad, Sarfaraz Ahmad Ejazi, Major Madhukar, Krishna Pandey, Mehebubar Rahman, Rama Prosad Goswami, Nahid Ali

**Affiliations:** 1 Infectious Diseases and Immunology Division, Indian Institute of Chemical Biology, Kolkata, India; 2 BD LifeSciences, Kolkata, India; 3 ICMR-Rajendra Memorial Research Institute of Medical Sciences (RMRIMS), Patna, India; 4 Department of Tropical Medicine, School of Tropical Medicine, Kolkata, India

**Keywords:** regulatory T cells (T regs), immunosuppression, visceral leishmaniasis, PBMCs, effector T cell (TE), immunology & infectious diseases

## Abstract

**Introduction:**

Regulatory T cells (Tregs) have been reported to control immune responses in microbial infections. However, their possible role in visceral leishmaniasis (VL) has not been well defined. To address this, we carried out extensive studies to investigate the frequency, phenotype and functions of kala-azar patients’ peripheral blood Tregs pre and post treatment.

**Methods:**

Fresh blood and peripheral blood mononuclear cells (PBMCs) were used to delineate the frequency and phenotype in VL through flow cytometry. Further for functional characterization, PBMCs of VL patients were depleted of CD25^+^ T cells and sorted Treg and T effector cells were co-cultured.

**Results:**

Tregs frequencies were significantly upregulated in the active VL patients compared to healthy controls and recovered individuals. Tregs characterized as CD4^+^CD127^-/low^CD25^high^ T cells expressed FoxP3 maximally. Isolated Treg cells from VL subjects displayed immunosuppression by inhibiting proliferation and IFN-γ production of effector cells. Moreover, Treg cells were functionally competent and exerted their suppressive role by inhibiting the IFN-γ production and proliferation of effector T cells. Interestingly, when analyzing Treg heterogeneity using the CD45RA marker, we observed an increased frequency of not only effector Treg subpopulations but also naïve and non-Treg cells in active VL patients.

**Conclusions:**

The present study characterizes the frequency, phenotype and function of CD4^+^CD127^-/low^CD25^high^Treg cells of kala-azar patients *ex vivo*. Our results suggest that functional effector Treg subpopulation elevated during active VL modulate effectors of immune response and induce immunosuppression. These together with naïve and non-Tregs cells constitute a defining feature of VL pathogenesis.

## Introduction

Visceral leishmaniasis (VL), or kala-azar, is a potentially devastating disease caused by *Leishmania donovani* and *Leishmania infantum*, with a global incidence of 50,000 to 90,000 new cases annually (1). A hallmark of active VL is severe cell-mediated immunosuppression, wherein peripheral blood mononuclear cells (PBMCs) fail to mount antigen-specific immune responses. This is evidenced by their inability to proliferate and produce IFN-γ in response to leishmanial antigens. However, this impairment is reversible, as patients regain their ability to produce *Leishmania*-specific IFN-γ and IL-12 following successful treatment, accompanied by a downregulation of IL-10 and TGF-β (2–5).

It appears that during disease, there is no inherent defect in *Leishmania*-specific T cell responses. Rather, the suppressed immune response observed in active VL suggests that immunity to *Leishmania* may be regulated by additional T cell-mediated mechanisms. The increased expression of anti-inflammatory cytokines and their correlation with parasite burden imply their involvement in the immunosuppression seen in VL patients (3, 4, 6, 7). However, the precise mechanisms responsible for this T-cell hyporesponsiveness or tolerance remain elusive.

Regulatory T cells (Tregs) have been reported to play a key role in maintaining peripheral immune tolerance and can modulate both innate and adaptive immune responses by reducing activation, proliferation, and cytokine production in effector and antigen-presenting cells (8, 9). Tregs originate from CD4^+^ T cells and are typically identified by the expression of the transcription factor FoxP3 or the IL-2 receptor α-chain (CD25) (10, 11). Additionally, the absence or low expression of the IL-7 receptor α-chain (CD127) has been proposed as a distinguishing marker for Tregs, given its inverse correlation with FoxP3 activity (12, 13). In humans, a combination of high CD25 and low or absent CD127 expression helps differentiate Tregs from effector T cells, which usually express high levels of CD127 (12, 14). However, these markers are not entirely specific to Tregs, as activated CD4^+^ T cells lacking suppressive activity can also express FoxP3 and CD25 or display a CD25^+^CD127^-^ phenotype (15, 16).

The significant role of CD4^+^ Treg cells in regulating immune responses in murine leishmaniasis has been well documented. In *Leishmania major* infection, natural Treg cells accumulate at the site of infection and modulate the function of effector cells in resistant C57BL/6 mice (17–21). Tregs can trigger disease reactivation and suppress effector memory responses when the balance between Treg and effector lymphocytes is disrupted during superinfection (21). IL-10-producing Tregs have also been implicated in vaccine failure using the LACK (*Leishmania*-Activated C-Kinase) antigen, and their depletion restored protective immunity (22). Moreover, Tregs that develop in response to *L. infantum* infection have been shown to promote parasite persistence and contribute to the establishment of chronic infection (23). Several studies in human cutaneous leishmaniasis have demonstrated a link between the presence of lesional natural Tregs and disease progression (24–27). However, studies focusing on Tregs in human VL patients are limited and have produced contradictory findings. One group, studying Tregs at disease-infiltrating sites, reported no enrichment of these cells in the spleen or blood (28, 29). In contrast, another group found enrichment of Tregs in the bone marrow and observed a correlation with parasite burden (30, 31).

In a previous study, we reported an increased frequency of Tregs (CD4^+^CD25^+^ cells) in the PBMCs of active VL patients, with a corresponding decline following successful therapy (3). In the present study, we used FoxP3 and CD127 in combination with CD25 to characterize the frequency, phenotype, and function of regulatory T cells in freshly isolated PBMCs from human VL patients before and after drug treatment. To better delineate effector Tregs from naïve and non-suppressive subsets, we also analyzed CD4^+^ T cell populations based on CD25 and CD45RA expression in VL patients.

## Results

### Characteristics of study cohort

Kala-azar patients (n = 68) confirmed to be VL by detection of amastigotes in splenic aspirates and/or by detection of antibodies against the recombinant antigen, rK39 were involved in the study. Blood samples were collected before and after treatment with conventional amphotericin B. Clinical parameters represent a significant improvement in white blood cell counts and haemoglobin levels after completion of treatment concomitant with reduced spleen size and no amastigotes in the spleen (**Table 1**). The patients were considered cured after completion of treatment with 20 mg/kg amphotericin B.

**Table 1:** Clinical characteristics of VL patients (mean ± SD).

Characteristic	AVL (n=68)	CVL (n=36)	HC (n=46)
Age (yrs)	26.93 ± 16.03	27.22 ± 16.03	34.89 ± 10.27
Male/Female	41/27	22/14	33/13
Body weight (Kg)	41.16 ± 13.71	38.13 ± 12.71	42.73 ± 11.01
Spleen size	8.43 ± 2.66	—	—
Duration of illness (months)	3.85 ± 3.34	—	—
Haemoglobin (g/dl)	7.91 ± 1.50	8.94 ± 2.22	11.26 ± 29.80
White blood cells	2965.54 ± 643.95	4674 ± 386	7732.50 ± 1617.71
Platelet count (1X 10^3^/ml)	135.19 ± 45.04	161. 47 ± 70.13	192.85 ± 69.48

AVL, Active Visceral Leishmaniasis; CVL, Cured Visceral Leishmaniasis; HC, Healthy Control.

### Identification of CD4^+^CD127^-/low^CD25^high^Treg cells

**Figure 1:**
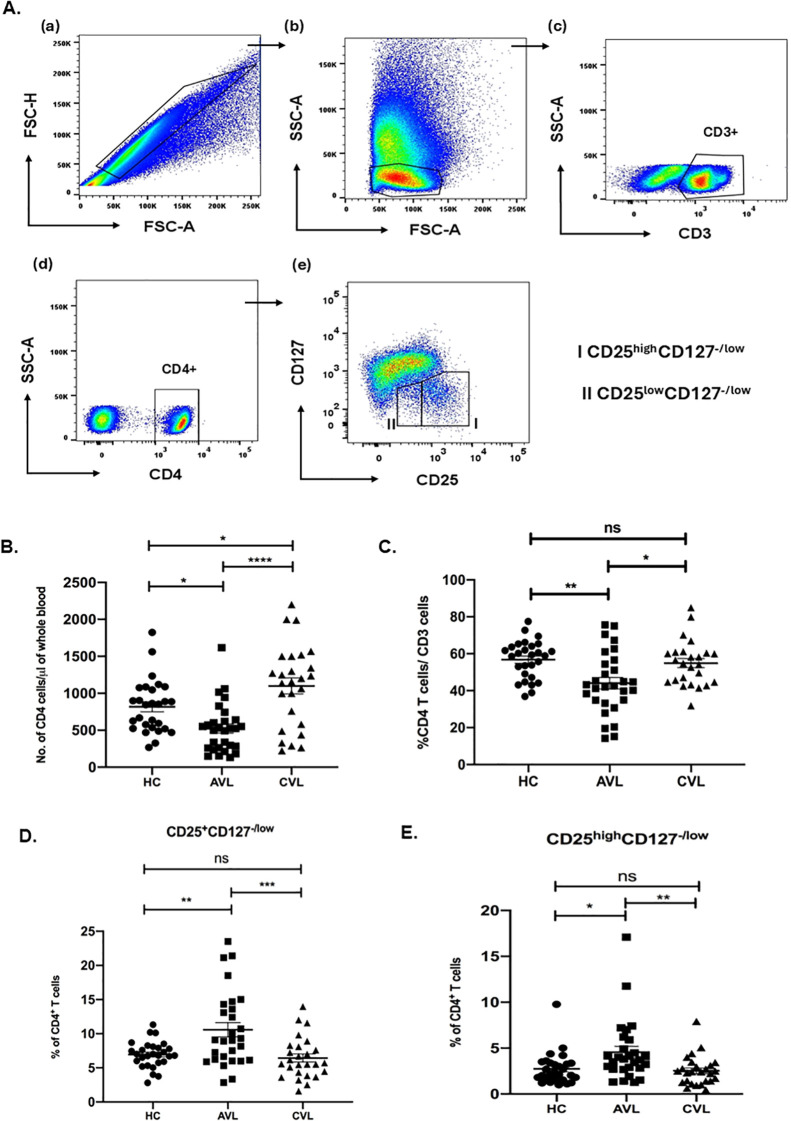
Increased Treg cell frequencies in active VL infection. Percentage of CD3, CD4 and Treg (CD4^+^CD127^-/low^CD25^+^ and CD4^+^CD127^-/low^CD25^high^) cells for active VL (AVL) (n = 29 except CD25^high^ Treg; n=30), cured VL (CVL) (n=26 except CD25^high^ Treg; n=25) and healthy controls (HC) (n=28) were determined through flow cytometry. Briefly, fresh blood cells were stained for CD3, CD4, CD127 and CD25 expression and gated on lymphocytes **(A)**. The T cell frequencies were represented as number of CD4 cells **(B)** and percentage of CD4^+^**(C)**, CD4^+^CD127^-/low^CD25^+^**(D)** and CD4^+^CD127^-/low^CD25^high^**(E)**. Each symbol represents percentage of T cells for one individual. Thick horizontal lines represent the mean values with standard error of mean (SEM) of each group. Total percentage of CD4^+^CD127^-/low^CD25^+^ and CD4^+^CD127^-/low^CD25^high^ cells of CD4^+^ were significantly upregulated in the active VL patients when compared with cured individuals and healthy controls. Values for *p* were calculated using Ordinary one-way ANOVA with a single pooled variance followed by Tukey’s multiple-comparisons test; *p* < 0.05 was considered significant. 0.1234 (ns), 0.0332 (*), 0.0021 (**), 0.0002 (***), <0.0001 (****).

Treg cells were identified by flow cytometry. Patients’ whole blood was stained *ex vivo* for CD3, CD4, CD127 and CD25. Cells were gated for lymphocytes using forward and side scatter properties. CD4^+^CD127^-/low^CD25^low^ and CD4^+^CD127^-/low^CD25^high^ cells were gated on CD3^+^CD4^+^cells as shown in **Figure 1A**. CD4^+^CD127^-/low^ cells with increased expression of CD25 appeared to the right from the major population as a tail and is described as CD4^+^CD127^-/low^CD25^high^ Treg cells.

### Frequency of Treg cells in blood of VL patients before and after treatment

To determine whether CD4^+^CD127^-/low^CD25^+^ Treg cells play a role in the immune suppression of VL patients, we compared the Treg cells frequencies present in the blood of active VL patients, cured individuals and healthy controls. Frequencies of the cells were determined through flow cytometry analysis using patients’ fresh blood *ex vivo*. Since VL patients are leucopenic (**Figure 1B**), we first determined the frequency of CD3, CD4 T cells among active VL, healthy controls and recovered individuals (**Figure 1C**). During active VL the percentage of CD4 cells were significantly lower, compared to cured VL and healthy controls.

Evaluation of CD4^+^CD127^-/low^CD25^+^ and CD4^+^CD127^-^CD25^high^ T cells, as a percentage of total CD4^+^ T cells, however, revealed a significantly higher percentage of CD4^+^CD127^-/low^CD25^+^Treg cells within the population of CD4^+^ T cells of VL patients (10.57 ± 1.04) (mean ± SEM) compared to healthy controls (6.94 ± 0.36) and cured VL individuals (6.44 ± 0.59) (**Figure 1D**). Likewise, percentage of CD4^+^CD127^-/low^CD25^high^ Treg cells of CD4^+^ were also significantly higher in VL patients as compared to healthy controls and recovered individuals (**Figure 1E**).

Our study exhibited that the percentage of both CD4^+^CD127^-/low^CD25^+^ and CD4^+^CD127^-^CD25^high^ T cells within the total CD4^+^ T cells were significantly higher in the active VL patients compared to cured and healthy controls.

### Depletion of CD25 cells from the PBMCs of active VL patients rescues leishmanial antigen specific lymphoproliferation and IFN-γ production

**Figure 2:**
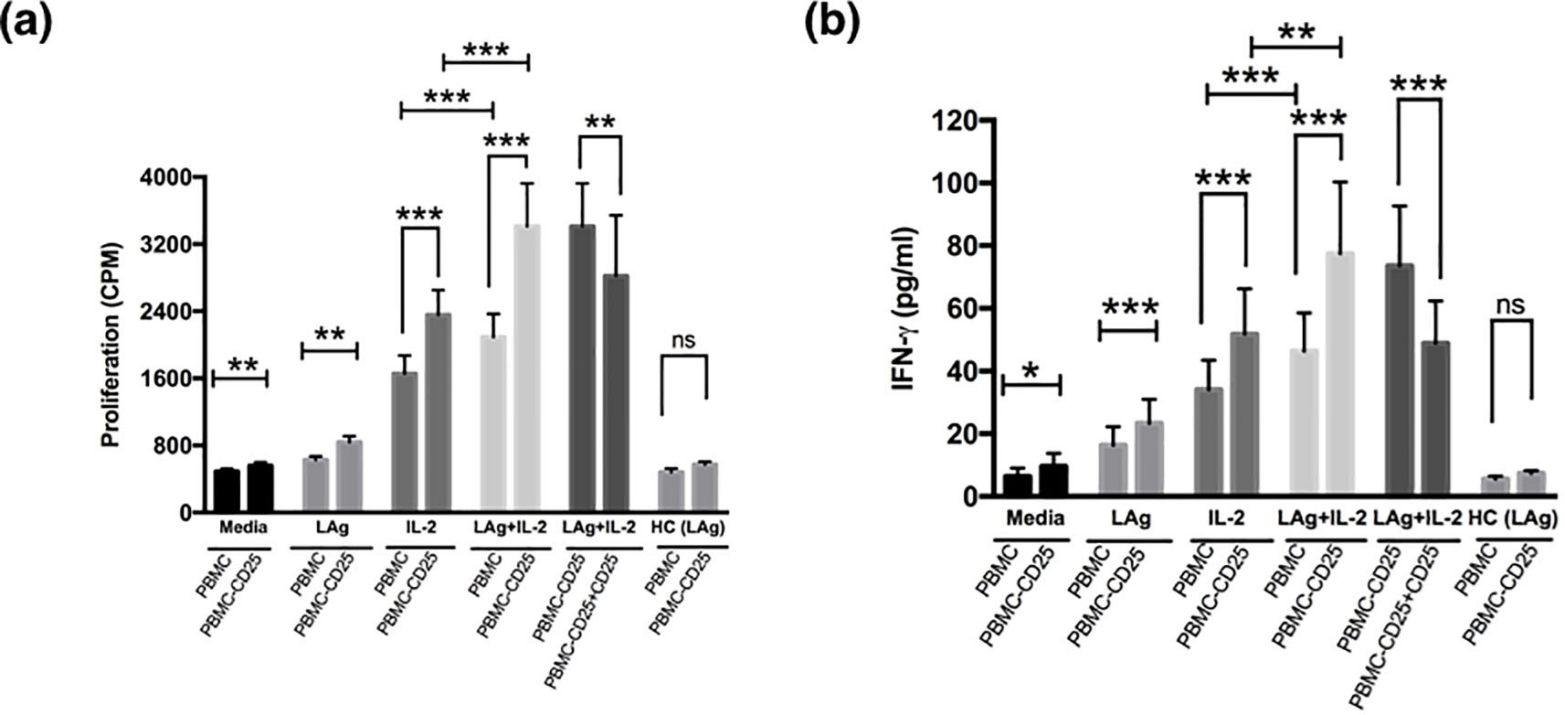
Treg cells suppress leishmanial antigen specific lymphoproliferation and cytokine production of active VL patients. **(a)** PBMCs and PBMCs depleted of CD25 cells (PBMC − CD25) of active VL patients were cultured (1× 10^5^ cells) for 5 days with media alone, LAg (12.5 µg/ml), only IL-2 or LAg plus rIL-2 (20 U/ml) where rIL-2 was added at day 3 of culture. For proliferation, cells were pulsed with 0.5 μCi/well [^3^H] thymidine and cultured for another 18 hours. Depletion of CD25 cells enhanced antigen-specific proliferation and further addition of CD25 cells to the depleted population (PBMC - CD25) at 1:1 ratio downregulated proliferation significantly. **(b)** Cells were cultured as described in **(a)** and supernatant was collected before the addition of thymidine and IFN-γ was detected through ELISA. Depletion of CD25 from PBMCs significantly upregulated IFN-γ production and reconstitution of CD25 cells to the depleted population (PBMC - CD25) at 1:1 ratio significantly downregulated IFN-γ production. LAg stimulated PBMCs and PBMCs depleted of CD25 cells (PBMC − CD25) of healthy controls (n=5) were also included in each figure. Bars represent mean ± SEM of proliferation and IFN-γ production of active VL patients (n=10). Values for *p* were calculated using Wilcoxon signed rank test; *p* < 0.05 was considered significant. 0.1234 (ns), 0.0332 (*), 0.0021 (**), 0.0002 (***).

To test if Treg cells mediate antigen-specific unresponsiveness, we examined the effects of CD25 cell depletion on lymphoproliferation and IFN-γ secretion by PBMCs when stimulated with leishmanial promastigote membrane antigen (LAg) alone or together with a low dose of recombinant IL-2 (rIL-2) (**Figure 2**). Contrary to whole cell soluble *Leishmania* antigen, or whole cell lysate/sonicate, LAg induces least nonspecific immune response in terms of lymphoproliferation and proinflammatory cytokine production from PBMCs of VL patients (3). PBMCs from VL patients responded very little to stimulation with LAg alone as there was very low lymphoproliferation and IFN-γ production (**Figures 2a, b**). LAg-specific proliferation and IFN-γ responses in VL PBMCs increased by removal of CD25 cells. Interestingly, stimulation with LAg together with rIL-2 (20 U/ml) for the last two days of culture showed significant elevation in lymphoproliferation and IFN-γ production in PBMCs depleted of CD25 cells when compared with their total PBMCs. Moreover, proliferation and IFN-γ production of CD25 depleted PBMCs reduced significantly in the presence of CD25 cells when reconstituted at the ratio of 1:1. Dampening of lymphoproliferation and cytokine production of effector T cells by Tregs was LAg-specific.

### CD4^+^CD25^+^Treg cells isolated from active and cured VL patients are functionally competent and suppress the cellular immune responses of CD4^+^CD25^-^ effector T cells stimulated with anti-CD3/anti-CD28

**Figure 3:**
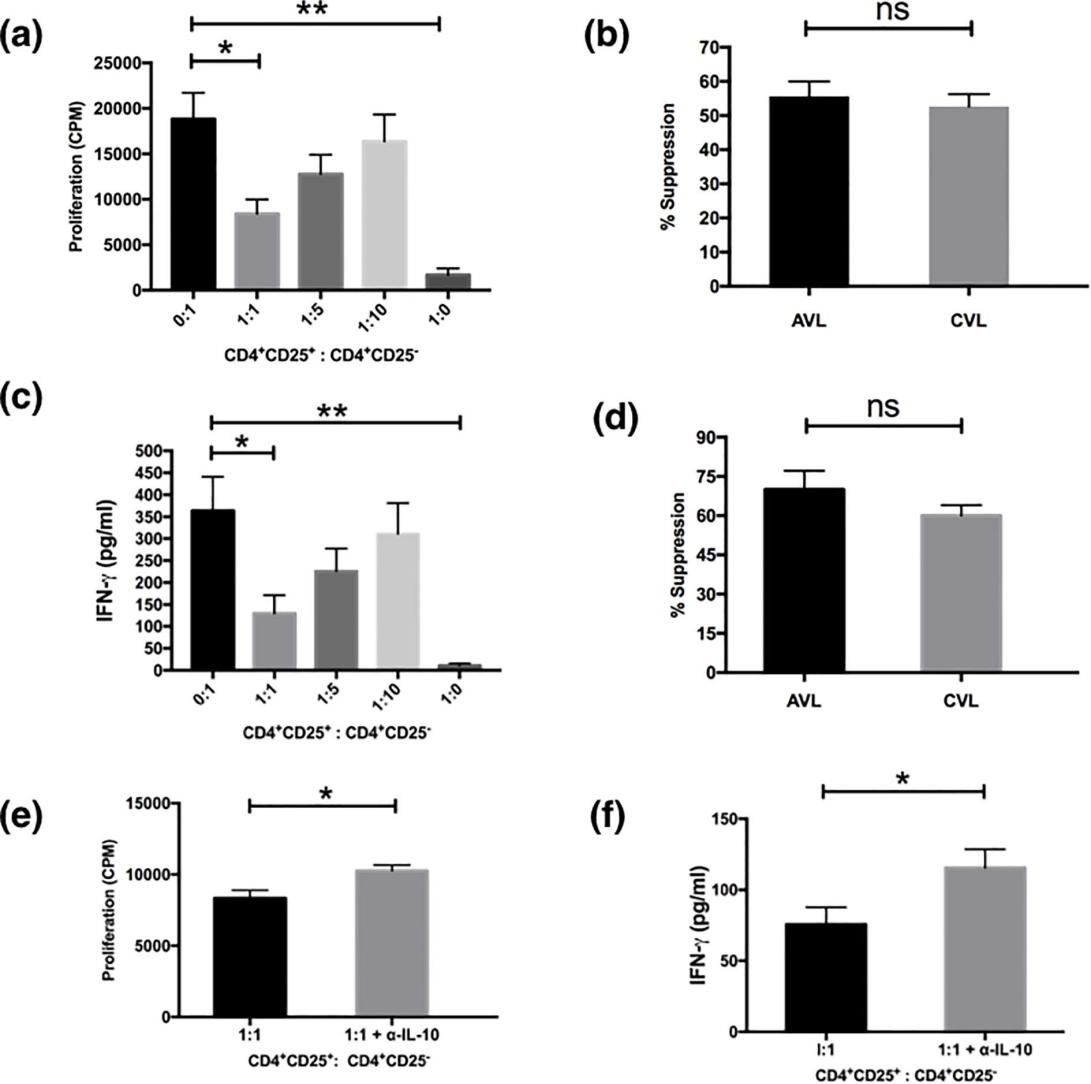
Treg cells from the PBMCs of VL patients suppress proliferation and cytokine production of effector cells. MACS sorted CD4^+^CD25^+^ cells and CD4^+^CD25^-^ cells of active VL patients (n=5) were cultured (1×10^5^ cells/well) in U - bottom tissue culture plates either alone or cocultured at ratios of 1:1, 1:5 and 1:10 and stimulated with soluble anti CD3 (1μg/ml) and anti CD28 (1μg/ml) for 5 days. For the last 18 hours the cultures were pulsed with 0.5 μCi/well of [^3^H]-thymidine. Cytokines were detected from the cell culture supernatants after 4 days of coculture through ELISA. **(a)** CD4^+^CD25^+^ cells suppressed the proliferation of CD4^+^CD25^-^ cells in a dose dependent manner. **(b)** Comparison of percentage suppression of proliferation by CD4^+^CD25^+^ cells from active and cured VL (n=5) subjects when cocultured with CD4^+^CD25^-^ effector cells at 1:1 ratio. **(c)** Detection of IFN-γ from the coculture experiments. Maximum IFN-γ was produced by CD4^+^CD25^-^ T cells which were reduced after coculture with CD4^+^CD25^+^ cells in a dose dependent manner. **(d)** Comparison of percentage suppression of IFN-γ production by CD4^+^CD25^+^ cells from active and cured VL subjects when cocultured with CD4^+^CD25^-^ effector cells at 1:1 ratio. Neutralization with anti-IL-10 in cocultured experiment (1:1 ratio) significantly restored the proliferation **(e)** and IFN-γ production **(f)** in active VL (n=6). Data represented as mean ± SEM for proliferation and cytokine analysis. Percentage suppression was calculated as follows: [1 - (mean CPM/cytokine of coculture wells)/(means CPM/cytokine of CD4+CD25- cells cultured alone)] × 100; where CPM indicates counts per minute. Values for *p* were calculated using Mann-Whitney *U* test; *p* < 0.05 was considered significant. 0.1234 (ns), 0.0332 (*), 0.0021 (**).

One hallmark of genuine Treg cells is their ability to suppress the activation of other cells. In the present study, we elucidated the function of Treg cells in active VL patients by measuring their potency to suppress the proliferation of CD4^+^CD25^-^ responder T cells proliferation (**Figure 3**). CD4^+^CD25^+^ and CD4^+^CD25^-^ T cells were separated from active and recovered VL subjects and co-cultured in different ratios of 0:1, 1:1, 1:5, 1:10 in the presence of soluble anti-CD3 (1µg/ml) and anti-CD28 (1µg/ml) antibodies. Proliferation was measured in terms of [^3^H] thymidine incorporation post 4 days of culture. It was observed that the proliferative capacity of CD4^+^CD25^-^ cells of active VL patients was highest when cultured alone (**Figure 3a**). However, CD4^+^CD25^+^ cells failed to show any proliferation in the presence of anti-CD3 and anti-CD28. When both the populations of CD4^+^CD25^+^ and CD4^+^CD25^-^ T cells were co-cultured in various ratios, a dose mediated suppression in proliferation of CD4^+^CD25^-^ cell population was observed, with the highest suppression observed at the ratio of 1:1. Similar results were also observed for the cured VL patients, and the level of suppression was comparable. At 1:1 ratio active VL patients showed (55.65 ± 4.33%) suppression whereas cured patients showed (52.65 ± 3.60%) (**Figure 3b**). Based on these findings, we conclude that Tregs from both active and cured VL exhibit comparable suppressive capacity ex vivo; however, active VL is marked by an increased Treg-to-effector T-cell ratio. Successful treatment normalizes this ratio and restores antigen-specific IFN-γ responses. This imbalance likely contributes to immunosuppression during VL.

The effect of the CD4^+^CD25^+^ T cells on cytokine secretion in the co-culture experiments was examined next (**Figure 3c**). We analyzed the supernatants taken from the cultures depicted in **Figure 3a** for IFN-γ. The levels of IFN-γ secretion mirrored the levels of proliferation when CD4^+^CD25^+^ and CD4^+^CD25^-^ T cells were co-cultured at various ratios. The highest level of IFN-γ was produced by CD4^+^CD25^-^ cells (**Figure 3c**). The addition of CD4^+^CD25^+^ T cells results in marked suppression in IFN-γ production of the effector CD4^+^CD25^-^ cells in a dose mediated manner with the maximum suppression at the ratio of 1:1 (*P*< 0.05) (**Figure 3c**). CD4^+^CD25^+^ T cells alone only had a basal level of IFN-γ upon the stimulation by anti-CD3 and anti-CD28 as reported earlier (11). CD4^+^CD25^+^ T cells of cured VL individuals also showed similar suppressive functions (data not shown), but the level of suppression at the active disease was higher (70.03 ± 7.14%) than that of the cured VL patients (60.05 ± 3.93%) although statistically they were not significant (**Figure 3d**). IL-10 is a well-known immunosuppressive cytokine to inhibit T cell proliferation secreted by regulatory and non-T cell populations. To establish the inhibitory factors responsible for the suppressive activity of CD25 T cells we investigated the role of IL-10. In order to see whether the suppression is IL-10 dependent or not, we neutralized the IL-10 in co-culture experiments. Although proliferation and IFN-γ production were restored significantly (*p<0.05)* by IL-10 neutralization the effect was only partial (**Figures 3e, f**). This suggests that regulation by CD4^+^CD25^+^ T cells is partially dependent upon IL-10.

### FoxP3 staining of CD4^+^CD127^-/low^CD25^high^ T cells in PBMCs of VL patients before and after treatment

**Figure 4:**
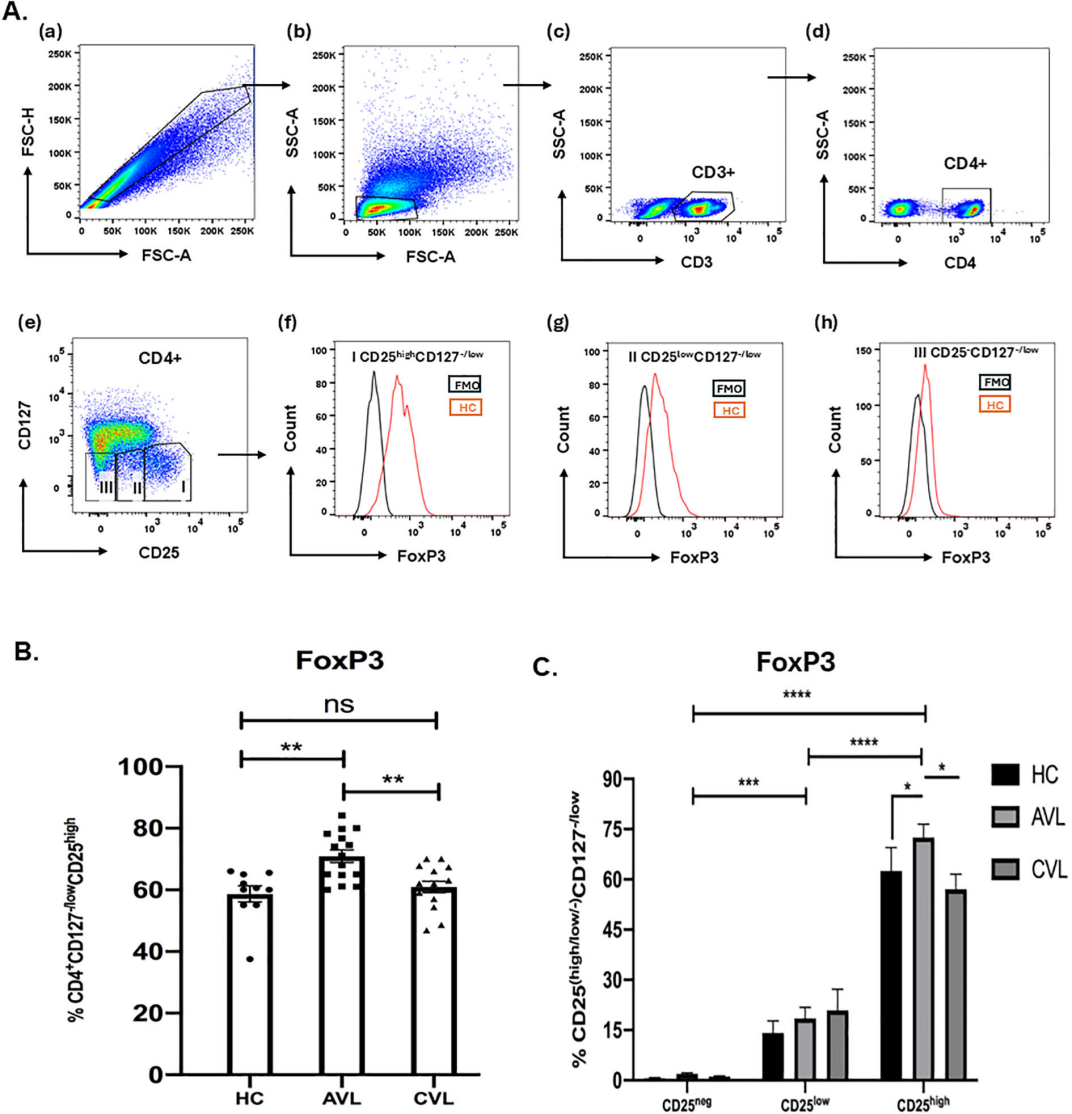
FoxP3 staining of CD4^+^CD127^-/low^CD25^+^ Tregs. Freshly isolated PBMCs from the blood of active VL, cured VL and healthy control, both for unpaired **(B)** (AVL; n = 15, CVL; n = 15, HC; n = 10) and paired samples **(C)** (AVL; n = 5, CVL; n = 5, HC; n = 7) were stained ex vivo for CD3, CD4, CD127 and CD25 followed by intracellular staining for FoxP3 T cells **(A)**. Total percentage of FoxP3 of the CD4^+^CD127^-/low^CD25^high^**(B)** and CD25^-^, CD25^low^ and CD25^high^ were determined through flow cytometry **(C)**. *P* values were calculated using Wilcoxon matched pairs signed rank test for paired samples and *p* < 0.05 was considered significant. Unpaired data **(B)** were analyzed using ordinary one-way ANOVA with a single pooled variance, followed by Tukey’s multiple-comparisons test. 0.1234 (ns), 0.0332 (*), 0.0021 (**), 0.0002 (***), <0.0001 (****).

Upon activation naive T cells often express CD25 and the CD25 cell population of active and cured VL patients might possess activated T cells along with Treg cells. FoxP3 is considered an appropriate marker to distinguish between activated and Treg cells (32). To control our CD25 based gating, staining of fresh PBMCs of active, recovered and healthy subjects for FoxP3 was done (**Figure 4**) so as to analyze the percentage of FoxP3 cells among variably expressing CD25 T cells. During the diseased state, frequency of CD4^+^CD127^-/low^CD25^high^ cells expressing FoxP3 (72.48±3.97%) (Mean±SEM) was higher in comparison to that of CD4^+^CD127^-/low^CD25^low^ (18.50±3.29) and CD4^+^CD127^-/low^CD25^-^cells (1.88±0.36) among paired samples (**Figure 4C**). Likewise in unpaired samples the percentage of Foxp3 cells among CD4^+^CD127^-/low^CD25^high^ was higher in active VL patients is (70.88± 2.04), compared to cured (60.94±1.82) and healthy subjects (58.65± 2.65) (**Figure 4B**). Collectively, these findings indicate that the gated CD4^+^CD127^-/low^CD25^high^ population in kala-azar comprises bona fide regulatory T cells rather than activated T cells; this phenotype is also observed in healthy individuals and cured patients.

### Phenotypic characterization of CD4^+^CD127^-/low^CD25^-^, CD4^+^CD127^-/low^CD25^low^ and CD4^+^CD127^-/low^CD25^high^ T cells of active VL patients, cured and healthy individuals

**Figure 5:**
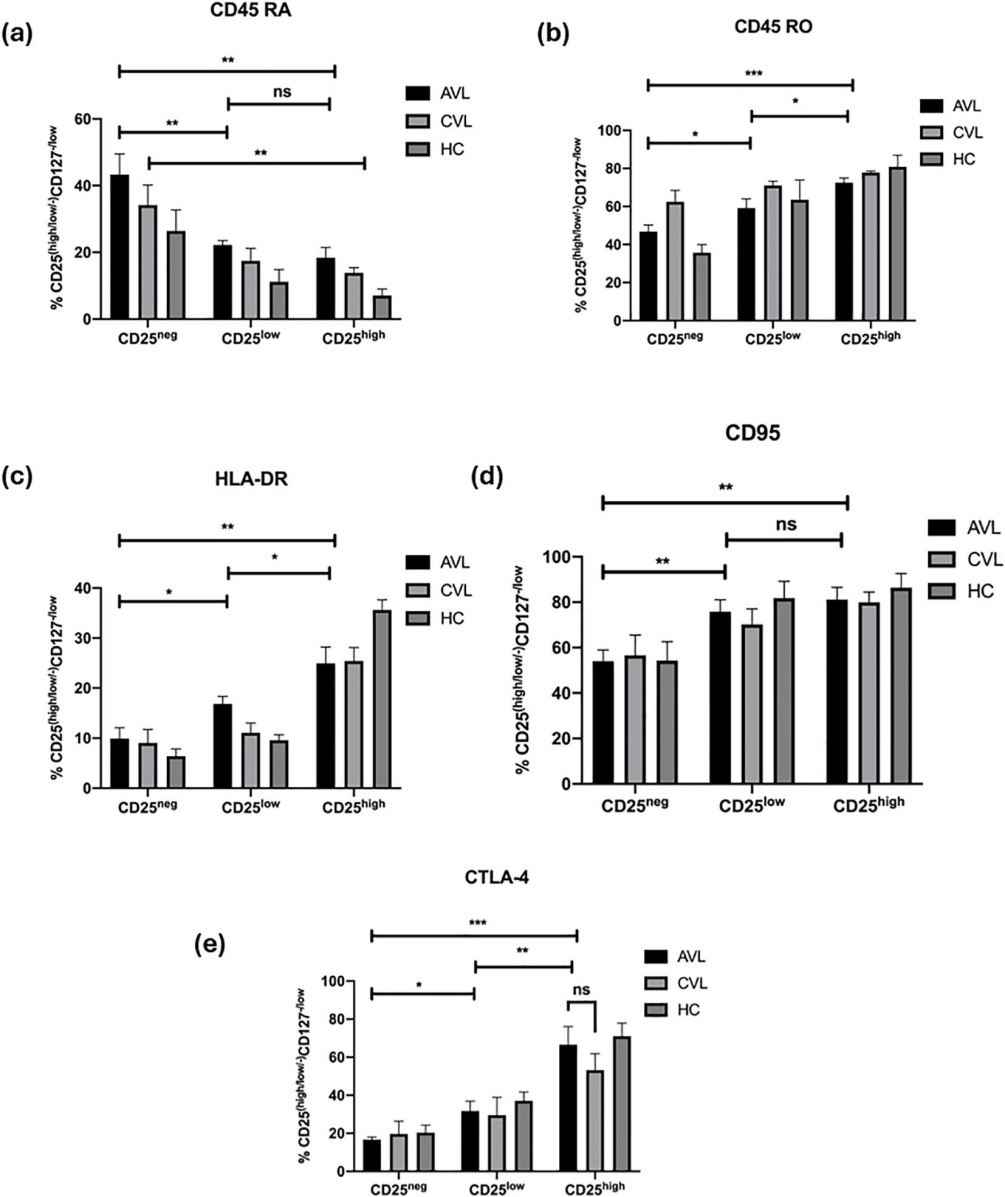
CD25^high^ Treg cells in VL infection show conventional phenotypes. Fresh whole blood of active VL (n=5), recovered (n=5) and healthy subjects (n=5) were stained ex vivo for CD3, CD4, CD127, CD25, and **(a)** CD45RA, **(b)** CD45RO, **(c)** HLA-DR, **(d)** CD95 and **(e)** CTLA-4 and analyzed through flow cytometry. CD25^-^, CD25^low^, and CD25^high^ cells were compared. The results are expressed as the mean ± SEM for the active VL (AVL), cured VL (CVL) and healthy control. *P* values were calculated using Wilcoxon matched pairs signed rank test for paired samples and *p* < 0.05 was considered significant. 0.1234 (ns), 0.0332 (*), 0.0021 (**), 0.0002 (***).

To decipher the phenotype of Treg cells with variable expression of CD25, stained *ex vivo* cells were analyzed using flow cytometry. The expression of surface and intracellular antigens (Ag) on these cells would help to understand their mechanistic insight and further characterize this regulatory population in kala-azar patients. The different levels of surface Ag expression like the memory marker CD45RO, marker for naïve T cells CD45RA, death receptor CD95, MHC-II (HLA-DR) and intracellular inhibitory receptor CTLA-4 were compared among the CD4^+^CD127^-/low^CD25^-^, CD4^+^CD127^-/low^CD25^low^ and CD4^+^CD127^-/low^CD25^high^ cell subsets in active, cured and healthy subjects (**Figure 5**, **Supplementary Figures 1**-**5**). During the diseased state, the frequency of CD4^+^CD127^-/low^CD25^high^ cells expressing CD45RO (72.52 ± 2.46%) (Mean ± SEM), CD95 (81.14 ± 5.43%), HLA-DR (24.94 ± 3.27%) and CTLA-4 (66.50 ± 9.56%) was significantly higher than the CD4^+^CD127^-/low^CD25^-^ cells (46.82 ± 3.49%), (54.02 ± 4.98%), (9.90 ± 2.18%), (16.70 ± 1.34%). In contrast CD45RA showed the opposite expression profile with higher percentage of CD4^+^CD127^-/low^CD25^-^ cells (43.32 ± 6.19) expressing this antigen than CD4^+^CD127^-/low^CD25^high^ cells (18.38 ± 3.09). Similarly, at the cured stage, there was significantly (*P* < 0.05) higher percentage of CD4^+^CD127^-/low^CD25^high^ cells expressing CD45RO, HLA-DR and CTLA-4 compared to CD4^+^CD127^-^CD25^-^ cells. A higher frequency of CD4^+^CD127^-/low^CD25^-^ cells expressed CD45RA (*P* < 0.05) at cure in comparison to CD4^+^CD127^-/low^CD25^high^ cells.

Collectively, the surface phenotype of the CD4^+^CD127^-/low^CD25^high^ cells in active VL patients did not differ much from that seen at recovery. In healthy controls, CD4^+^CD127^-/low^CD25^high^ cells are CD45RO^high^, CD95^high^, HLA-DR^high^ and CTLA-4^high^. Furthermore, we observed that the naïve T cell marker, CD45RA was found in the CD4^+^CD127^-/low^CD25^-^ subset and not in the CD4^+^CD127^-/low^CD25^high^ T cells. These results indicate that CD4^+^CD127^-/low^CD25^high^ T cells of active and cured VL patients are regulatory T cells and not activated T cells, having a phenotype largely similar to natural Tregs of healthy controls.

### The frequency of effector Treg subpopulations is increased in the peripheral blood of VL patients

**Figure 6:**
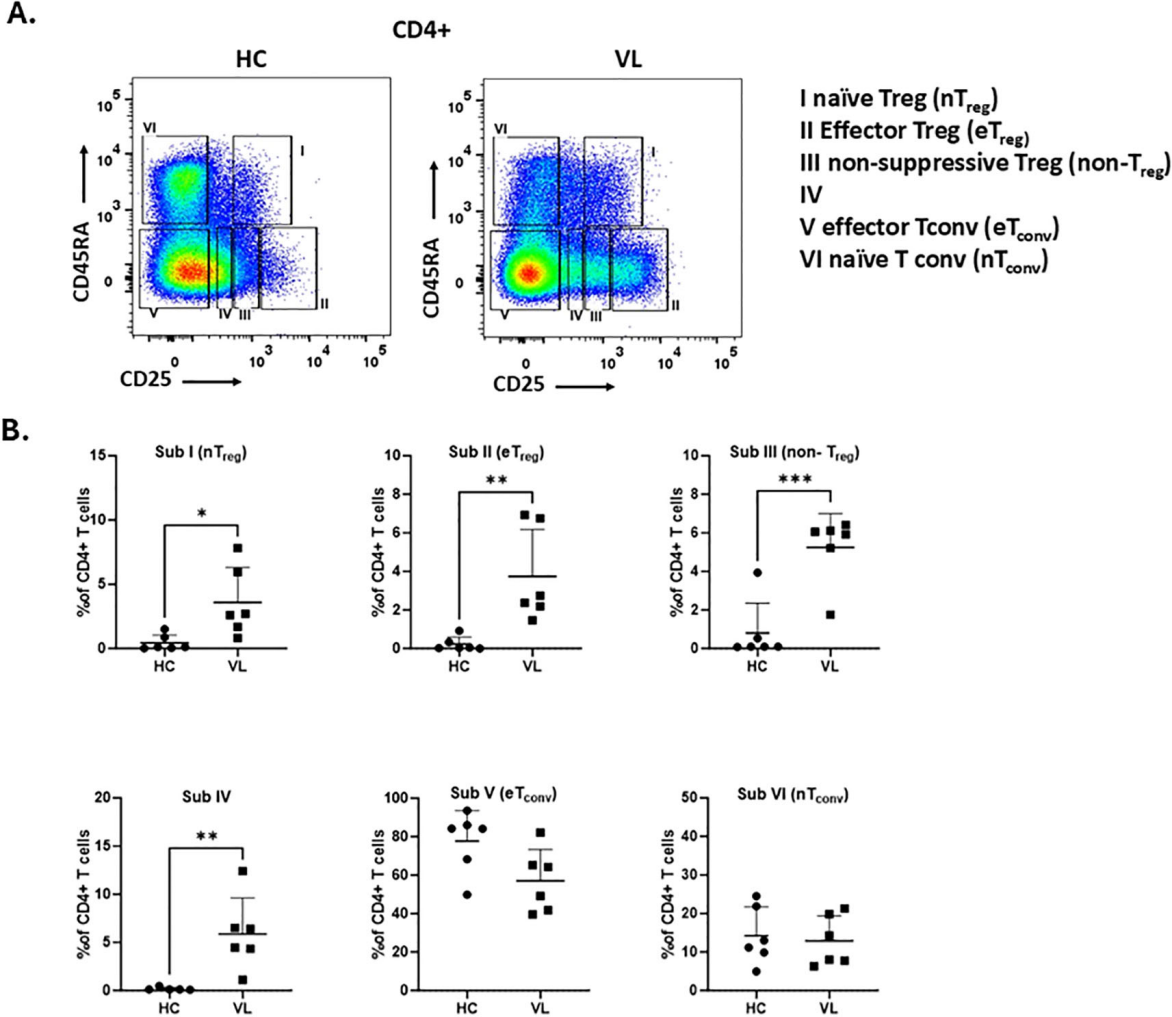
The frequency of effector T_reg_ subpopulations is increased in the peripheral blood from VL patients. **(A)** Gating strategy to define six CD4^+^ T cell subpopulations based on differential expression of CD25 and CD45RA.: CD25^int^CD45RA^+^ cells (Subgroup I, naïve Treg), CD25^high^CD45RA^−^ cells (Subgroup II, effector Treg), CD25^int^CD45RA^−^ cells (Subgroup III, non-suppressive Treg), CD25^low^CD45RA^−^ cells (Subgroup IV), CD25^−^CD45RA^−^ cells (Subgroup V, effector Tconv), and CD25^−^CD45RA^+^ cells (Subgroup VI, naïve Tconv). **(B)** Frequency of each subgroup within CD4^+^ T cells from PBMCs of healthy controls (HC, n = 6) and VL patients (n = 6). Statistical differences between HC and VL were assessed using t-test and shown as p-values. 0.0332 (*), 0.0021 (**), 0.0002 (***).

Since CD45RA can also differentiate between antigens experienced Treg (e.g. CD45RA^-^) and naive Treg (e.g. CD45RA^+^) cells (32), we introduced this marker to study the heterogenicity of Treg cells in the Treg subgroups (**Figure 6A**). The subgroups include CD25^int^CD45RA^+^ cells (Subgroup I, naïve/resting Treg), CD25^high^CD45RA^-^ cells (Subgroup II, activated/effector Treg), CD25^int^CD45RA^-^ cells (Subgroup III, non-suppressive Treg), CD25^low^CD45RA^-^ cells (Subgroup IV), CD25^-^CD45RA^-^cells (Subgroup V, effector Tconv), and CD25^-^CD45RA^+^ cells (Subgroup VI, naïve Tconv). Using these groupings we found the frequency of effector Treg cells (Subgroup II, CD25^high^CD45RA^-^) was markedly increased in VL patients compared to healthy controls (**Figure 6B**). Along with eTreg, naive Treg (Subgroup I, CD25^int^CD45RA^-^) cells, non-suppressive Treg (Subgroup III, CD25^int^CD45RA^-^) cells as well as Subgroup IV (CD25^low^CD45RA^-^) were also significantly increased in VL patients. In contrast, the effector Tconv cells (Subgroup V, CD25^-^CD45RA^-^) was decreased in VL patients, though not significantly, compared to healthy controls, and there was no difference in naive Tconv cells (Subgroup IV, CD25^-^CD45RA^+^) of healthy controls versus VL patients. These data suggest an overall increase of CD25 expressing cells ranging from low to high in the peripheral blood of VL patients.

## Discussion

Treg cells play a pivotal role in maintaining immunological self-tolerance, preventing autoimmune diseases, and suppressing chronic inflammatory conditions (33). However, their role in human VL pathogenesis remains debatable (28–31). Our current understanding on Tregs and immunosuppressive mediators in VL is primarily derived from experimental mouse models (15–18, 20). Evidence from studies on chronic infectious diseases suggests a role of natural Tregs in the modulation of protective immune response (34). Building on our previous findings (3), we sought to address the controversy surrounding Tregs during *Leishmania* infection by analyzing their frequency, phenotype and function in a large cohort of well-characterized VL patients. Our data support the critical involvement of Tregs in disease progression revealing a strong association between increasing Treg/Teff ratios and advancing disease severity. Treg cells of infected kala-azar patients displayed frequencies and phenotypes comparable to those of cured individuals and healthy controls. Depletion of CD25 cells from PBMCs restored their antigen-specific proliferation and IFN-γ production. In the Media condition, we observed a little but significant increase in proliferation and IFN-γ compared with undepleted PBMC. We interpret this as release from Treg-mediated suppression. Tregs limit spontaneous T-cell activation by consuming IL-2 and reducing APC costimulation. After CD25 removal which primarily targets the Treg, basal signals present in PBMC cultures—tonic/self-peptide–MHC stimulation and homeostatic cytokines in medium—are sufficient to drive bystander proliferation and IFN-γ from T cells even without exogenous stimulus. Consistent with this mechanism, adding IL-2 further augments these responses, indicating that relief of IL-2 competition contributes to the effect Moreover, CD4^+^CD25^+^ T cells isolated from VL patients displayed immunosuppressing activity inhibiting both proliferation and IFN-γ production by effector T cells in both antigen-specific and non-specific contexts, similar to observations in cured individuals. Interestingly, effector Treg cell subset defined as CD45RA^-^CD25^high^ showed a marked increase in VL patients. In addition, we observed increased frequencies across all subsets including Subgroup I (nTreg), Subgroup III (non-Treg), and Subgroup IV with higher CD25 expression in VL patients than in controls. These findings suggest that the elevated frequency of CD25-expressing cells in the peripheral blood may contribute to the immune-suppression associated with VL pathogenesis.

Human VL is characterized by suppressed antigen-specific T cell proliferation and IFN-γ production, alongside elevated levels of immunosuppressive cytokines in patient PBMCs. Successful treatment restores T cell responses to leishmanial antigens accompanied by proliferation and IFN-γ production, and a concomitant decrease in suppressive cytokines (3). However, the mechanisms regulating CD4 effector T cell function during disease remains unclear. One group found no expansion of CD4^+^CD25^+^Foxp3^+^ Tregs in the peripheral blood or lesional tissue (spleen) of active VL patients and instead implicated CD4^+^CD25^-^FoxP3^-^ T cells in VL pathogenesis (28, 29). In contrast, another group reported enrichment of CD4^+^CD25^+^Foxp3^+^ Treg cells in the bone marrow of active VL patients (30, 31). Our earlier findings indicated an increased frequency of natural Tregs (CD4^+^CD25^high^) correlating with parasite burden in peripheral blood (3, 7). Consistent with reports of leucopenia in VL patients we also found reduced CD4^+^ T cells in the blood of active VL patients compared to healthy, and cured individuals (35). For Treg identification we employed CD25 (IL-2 receptor α chain) marker as previously reported by Sakaguchi et al. (36). However, CD25 expression alone is insufficient to identify Treg cells since it is also expressed at low levels in activated conventional T cells (T_conv_). Treg cells, in contrast, express low levels of CD127 (IL-7 receptor α chain) (14, 37). Using a combination of CD25 and CD127 we identified significantly elevated populations of CD4^+^CD127^-/low^CD25^+^ and CD4^+^CD127^-/low^CD25^high^ Treg cells in active VL patients compared to cured and healthy controls.

Functional analysis CD4^+^CD25^+^ Treg cells demonstrated their immunosuppressive capacity. Co-cultures of CD4^+^CD25^+^ T cells and CD4^+^CD25^-^ effector T cells revealed that the former suppressed proliferation and IFN-γ production in response to anti-CD3 and anti-CD28 in a dose-dependent manner. Notably, CD4^+^CD25^-^ Treg cells from active VL patients exhibited the highest levels of proliferation and IFN-γ production, confirming that suppression is not due to intrinsic defects in the CD4^+^ T cells, but rather due to CD4^+^CD127^-/low^CD25^+^ Treg population. Isolated CD4^+^CD25^+^ T cells from VL patients were functionally competent to polyclonal stimulation and produced negligible IFN-γ. Targeted depletion of CD25^+^ cells from PBMCs restored *Leishmania*–specific proliferation and IFN-γ production, affirming the regulatory role of Tregs. These findings contrast with a previous report by Nylen et al. which found no restoration of antigen-specific responses following CD25^+^ cell depletion (29).

To determine whether suppressive Treg cells expressed FoxP3, a hallmark of Treg identity, we analyzed CD25 T cells and found maximal FoxP3 expression in CD4^+^CD127^-^CD25^high^ subsets. An increase in this population during active disease followed by a decline after treatment further confirms their regulatory role. In post kala-azar dermal leishmaniasis (PKDL) FoxP3 and IL-10 expression in lesional tissues correlate with parasite burden (38). Similar elevations of CD4^+^CD25^+^ T cells in chronic human infections (39–41), including HIV (42–44), HBV (45, 46), and fungal disease (47) have been implicated in disease persistence.

Phenotypic analysis of circulating CD4^+^CD127^-^CD25^+^ T cells from VL patients and cured subjects revealed profiles largely consistent with those of healthy individuals (48). Importantly, CD4^+^CD127^-^CD25^high^ T cells in VL patients expressed CD45RO (memory marker) at higher frequencies, and CD45Ra (naive marker) at lower levels compared to CD25^neg^ effector cells. CD4^+^CD127^-^CD25^high^ cells also showed elevated expression of CD95 (Fas receptor) indicative of increased susceptibility to apoptosis (48). The proportion of cells expressing HLA-DR, CD45RO and CD95 were high among CD25^high^ T cell subsets than CD25^neg^ subsets a pattern similar to that observed in cured and healthy individuals. CTLA-4, a negative regulator of T cells (49–52), was also found to be upregulated in active VL patients. Among the phenotypically defined human Treg subsets CD45RA^+^HLADR^-^, CD45RA^-^HLA-DR^-^, and CD45RA^-^HLA-DR^+^, FoxP3 and CTLA-4 expression was predominantly associated with CD45RA^-^HLA-DR^+^ population (53), consistent with our findings.

Identifying human Tregs remains challenging due to overlapping features with activated T_conv_, including low-level FoxP3 (32). Our analysis showed maximal CD45RA expression in CD25^neg^ cells and minimal expression in CD25^high^ cells. Examination of Treg subsets among CD4^+^ T cells revealed a significant increase in effector Tregs (CD25^high^CD45RA^-^) in VL patients. In contrast, conventional CD4^+^ T cells showed reduced or unchanged CD25 expression. These results suggest that increased CD25 expression correlates with elevated frequencies of effector, naive, and non-Treg cells, constituting a defining feature of VL pathogenesis.

An interesting study would be to enroll patients with delayed parasitological clearance or clinical non-response, a group which was not observed with our first line regimen, to provide insight whether baseline or persistent Treg elevation predicts treatment failure. Our Treg phenotyping leveraged a validated core panel (CD45RA/RO, HLA-DR, CD95, CTLA-4, CD127/FOXP3) to robustly define naïve and activated subsets across treatment. Building on this foundation, future panels will incorporate CD39, CD73, PD-1, TIGIT, LAG-3, and TIM-3 to broaden functional and activation-state resolution and delineate regulatory mechanisms with greater precision. In the Treg–Teff co-cultures, we used anti-CD3/anti-CD28 stimulation to quantify intrinsic, APC-independent Treg suppressive capacity in a standardized setting. While inclusion of autologous monocytes pulsed with LAg would enable assessment of antigen-specific suppression (e.g., IFN-γ attenuation), limited cell yields, and inter-donor APC variability prevented systematic implementation across the cohort. Future studies will integrate LAg-pulsed APC co-cultures to delineate antigen-specific Treg function.

Taken together, integration of frequency, phenotypic, and functional data shows that both natural and effector Tregs expand in active VL, driving a higher Treg/Teff ratio that normalizes with treatment; these bona fide Tregs remain functionally suppressive in both active disease and cure, defining a regulatory signature of pathogenesis. Prospective longitudinal studies with standardized immune profiling and antigen-specific assays in paired blood and tissue are needed to validate this axis as a biomarker and to guide adjunctive immunotherapies that safely rebalance protective immunity.

## Methods

### Study cohort

Sixty-eight patients (males and females, and HIV negative) with clinical diagnosis of VL admitted to School of Tropical Medicine, Kolkata, West Bengal and Rajendra Memorial Research Institute of Medical Sciences (RMRIMS), Patna were enrolled for this study. VL was diagnosed by usual clinical presentations like prolonged fever, hepatosplenomegaly, anaemia, pancytopenia, and confirmed by rK39 strip test and demonstration of *Leishmania* parasites in the splenic or bone marrow aspirates. The patients were treated with amphotericin B deoxycholate (AmB) (Sarabhai Piramal Pharmaceuticals, India). AmB was administered by intravenous drip in dextrose solution on alternate days. Total dosage of the drug was 20 mg/kg body wt., starting with a low dose (0.1 mg), gradually increasing up to 1 mg/kg/day, which were continued till the completion of the full dose. Heparinized blood samples from the patients were collected before the initiation and after the completion of treatment. Blood samples were also collected from 46 healthy donors from the CSIR-Indian Institute of Chemical Biology, Kolkata, India (non-endemic for VL) as controls. Fresh peripheral blood mononuclear cells (PBMCs) from the patients and healthy donors were used for invitro cell culture assays.

### Ethics statement

The study was approved by the Ethical Committee on Human Subjects at CSIR- Indian Institute of Chemical Biology. Written informed consents were obtained from all patients and donors for blood sampling.

### Leishmanial antigen preparation

Leishmanial antigen was prepared from *L. donovani* promastigotes in their stationary phase from third or fourth passage. Cells in culture media were washed in chilled 20 mM phosphate-buffered saline (PBS, pH 7.4) and suspended in 5 mM cold Tris-HCl buffer (pH 7.6). The suspension was transferred to test tube and gone through six times vertexing for 2 min each with a 10 min interval followed by centrifuged at 2310 ×*g* for 10 min. The cell pellet obtained was resuspended in the same buffer and sonicated 3 times for 1 min pulse and 1 min interval. Subsequently, the suspension was centrifuged at 5190 ×*g* for 30 min and the supernatant containing leishmanial protein, LAg was isolated and stored at -70°C. From a 1 g of cell pellet approximately 12 mg of protein was obtained, as assayed by Lowry et al. (54).

### Flow cytometric analysis of surface and intracellular phenotype

Phenotypic study of CD4^+^CD127^-/low^CD25^+^ T cells was performed on freshly obtained heparinized whole blood samples from study subjects by direct flow cytometry. The following human mAbs (BD) were used for immunostaining: CD3-APC-Cy7, CD4-Qdot 655, CD25-Pacific Blue, CD127-PE-Cy7, CD45RA-APC (HI100), CD45RO-APC (UCHL1), CD95-APC (DX2), HLA-DR-APC (G46-6). Multiple panels were used for this phenotypic analysis. Freshly collected 100 μl of anti-coagulated whole blood samples was incubated with 20 μl of appropriate mAbs for 30 minutes in the dark at room temperature (RT). RBC were lysed by incubating with FACS™ lysing solution (BD) for 12 mins at RT followed by centrifugation at 300 × g. Supernatant was decanted, and cells were washed with 0.02 M PBS. Finally, the cells were taken in 0.02 M PBS containing 1% FCS for FACS analysis.

For detection of intracellular CTLA-4 and FoxP3, PBMCs were isolated from whole blood through density sedimentation on Histopaque-1077 (Sigma-Aldrich). PBMCs were first incubated with mAbs against surface markers CD3, CD4, CD25 and CD127 as described above. Cells were then washed with FACS buffer (0.02 M PBS, 1% FCS and 0.01% sodium azide), fixed and permeabilized with Cytofix/Cytoperm (BD) reagent for 20 min at 4°C and washed again with 0.1% saponin in FACS buffer. Intracellular staining was performed with CTLA-4 and FoxP3 for 45 min at 4°C. Cells were then washed and taken in 500 μl PBS containing 1% FCS for analysis. Cell acquisition and analysis were performed using FACS Canto using FlowJo software on at least 1, 00,000 events. Gating was restricted to the population of lymphocytes according to their light scattering properties.

### Cell isolation

Isolation of CD4^+^CD25^+^ and CD4^+^CD25^-^ cells were quickly carried out from fresh PBMCs of patients obtained through density sedimentation on Histopaque-1077 (Sigma-Aldrich). Briefly, negative selection of CD4^+^ T cells from total PBMC were performed using the CD4^+^ isolation kit (Miltenyi Biotec, BergischGladbach, Germany). Non-CD4^+^ T cells were first incubated in a biotin antibody cocktail (10 μl per 10^7^ total cells) for 10 mins at 4°C and then magnetically labeled with anti-biotin microbeads (20 μl per 10^7^ total cells) followed by another incubation of 15 mins at 4°C, the cells were then washed and depleted over MACS MS column. The untouched CD4^+^ T cells thus obtained was then labeled with anti-CD25 microbeads (10 μl per 10^7^ CD4^+^ cells), incubated for 15 mins at 4°C and eventually subjected to positive selection according to manufacturer’s instructions (Miltenyi Biotec). Among CD4^+^CD25^+^ T cells, yield of positive selection was ≥ 94% pure, of which ~ 65% cells expressed FoxP3 as determined by flowcytometry. The negative selection, on the other hand, yielded CD4^+^CD25^-^ T cells. Both these cellular subsets were used for co-culture experiments.

### Cocultures and proliferation assays

To study the regulatory function of Treg cells, freshly purified CD4^+^CD25^+^ T cells from infected and cured VL patients were cultured with their respective CD4^+^CD25^-^effector cells, at a ratio of 1:1, 1:5, 1:10, in 96-well U-bottom tissue culture plates (Nunc), in the presence of soluble anti CD3 (HIT3a) (1μg/ml) and anti CD28 (CD28.2) antibodies (1μg/ml) at 37°C and 5% CO_2_. To assess the role of IL-10 in the suppressive activity of theCD4^+^CD25^+^ T-cell population, CD4^+^CD25^-^ lymphocytes were co-cultured with CD4^+^CD25^+^ cells (1:1) from infected patients in the presence of 10 µg/ml neutralizing anti–IL-10 mAb (BD). On day 4, the culture supernatants were collected for IFN-γ cytokine analysis and 0.5 μCi/well [3H] thymidine was added for the final 18 hours of culture performed in triplicate. Total PBMCs, CD25-depleted PBMCs and CD25-depleted PBMCs plus CD25 cells (1:1) (1 × 10^5^/well) of the infected patients were also stimulated with LAg (12.5 µg/ml) for 3 days. For cytokine analysis and proliferation, the plates were cultured under the same conditions for 5 days, with replenishment of fresh medium containing 20 U/ml recombinant human (rh) IL-2 (BD) at day 3. For the last 18 h the cultures were pulsed with 0.5 μCi/well [3H] thymidine. Incorporation of [3H] thymidine was measured using a liquid scintillation counter. Data are represented as counts per minute (cpm). Percentage suppression of cellular proliferation and cytokine production of CD4^+^CD25^-^ T cells when cocultured with CD4^+^CD25^+^ T cells at 1:1 ratio was calculated using the formula: [1 – (cpm in the presence of CD4^+^CD25^+^ T cells)/(cpm in the absence of CD4^+^CD25^-^ T cells)] x 100%.

### Statistical analysis

Statistical analyses were performed using GraphPad Prism (GraphPad Software, San Diego, CA, USA). For two-group comparisons, we used the Wilcoxon matched-pairs signed-rank test (paired) and the Mann–Whitney U test (unpaired). For comparisons involving three or more groups, we applied Ordinary one-way ANOVA with a single pooled variance followed by Tukey’s multiple-comparisons test. P values < 0.05 were considered statistically significant.

## Data Availability

The original contributions presented in the study are included in the article/**Supplementary Material**. Further inquiries can be directed to the corresponding author.

## Supplementary material

Supplementary Figure 1 Gating strategies for identification of different subsets of Treg and CD45RA staining. Fresh peripheral blood was stained ex vivo for CD3, CD4, CD127, CD25 and CD45 RA for multiparametric analysis through FACS. CD3^+^ and CD4^+^ cells were gated from the single cell population of T lymphocytes **(a–d)**. CD3^+^CD4^+^ population was gated with low expression of CD127 (CD3^+^CD4^+^CD127^-/low^) and variable expression of CD25, CD25^-^, CD25^low^ and CD25^high^**(e)**. Percentage of CD45 RA positive cells in the CD25^high^, CD25^low^, and CD25^-^ cell populations are shown as histograms **(f–h)**. Fluorescence minus one (FMO) staining was used both for gating CD127 and CD45 RA. Gating strategy is illustrated using a representative healthy control sample.

Supplementary Figure 2 Gating strategies for identification of different subsets of Treg and CD45RO staining. Fresh peripheral blood was stained ex vivo for CD3, CD4, CD127, CD25 and CD45 RO for multiparametric analysis through FACS. CD3^+^and CD4^+^ cells were gated from the single cell population of T lymphocytes **(a–d)**. CD3^+^CD4^+^ population was gated with low expression of CD127 (CD3^+^CD4^+^CD127^-/low^) and variable expression of CD25, CD25^-^, CD25^low^ and CD25^high^**(e)**. Percentage of CD45 RO positive cells in the CD25^high^, CD25^low^, and CD25^-^ cell populations are shown as histograms **(f–h)**. Fluorescence minus one (FMO) staining was used both for gating CD127 and CD45 RO. Gating strategy is illustrated using a representative healthy control sample.

Supplementary Figure 3 Gating strategies for identification of different subsets of Treg and HLA-DR staining. Fresh peripheral blood was stained ex vivo for CD3, CD4, CD127, CD25 and HLA-DR for multiparametric analysis through FACS. CD3^+^and CD4^+^ cells were gated from the single cell population of T lymphocytes **(a–d)**. CD3^+^CD4^+^ population was gated with low expression of CD127 (CD3^+^CD4^+^CD127^-/low^) and variable expression of CD25, CD25^-^, CD25^low^ and CD25^high^**(e)**. Percentage of CD95 positive cells in the CD25^high^, CD25^low^, and CD25^-^ cell populations are shown as histograms **(f–h)**. Fluorescence minus one (FMO) staining was used both for gating CD127and HLA-DR. Gating strategy is illustrated using a representative healthy control sample.

Supplementary Figure 4 Gating strategies for identification of different subsets of Treg and CD95 staining. Fresh peripheral blood was stained ex vivo for CD3, CD4, CD127, CD25 and CD95 for multiparametric analysis through FACS. CD3^+^and CD4^+^ cells were gated from the single cell population of T lymphocytes **(a–d)**. CD3^+^CD4^+^ population was gated with low expression of CD127 (CD3^+^CD4^+^CD127^-/low^) and variably expression CD25, CD25^-^, CD25^low^ and CD25^high^**(e)**. Percentage of HLA-DR positive cells in the CD25^high^, CD25^low^, and CD25^-^ cell populations are shown as histograms **(f–h)**. Fluorescence minus one (FMO) staining was used both for gating CD127and CD95. Gating strategy is illustrated using a representative healthy control sample.

Supplementary Figure 5 Gating strategies for identification of different subsets of Treg and CTLA-4 staining. Fresh PBMCs were stained ex vivo for CD3, CD4, CD127 and CD25 followed by intracellular staining for CTLA-4 T cells. CD3^+^and CD4^+^ cells were gated from the single cell population of T lymphocytes **(a–d)**. CD3^+^CD4^+^ population was gated with low expression of CD127 (CD3^+^CD4^+^CD127^-/low^) and variable expression of CD25, CD25^-^, CD25^low^ and CD25^high^ expression **(e)**. Percentage CTLA-4 positive cells in the CD25^high^, CD25^low^, and CD25^-^ cell populations are shown as histograms **(f–h)**. Fluorescence minus one (FMO) staining was used both for gating CD127 and CTLA-4. Gating strategy is illustrated using a representative healthy control sample.
