# Supplementary figures and images for: Functional and phenotypic analysis of CD4 T cell dynamics in peripheral blood of human visceral leishmaniasis patients confers increased frequencies of CD25 expressing regulatory T cells that contribute to disease pathogenesis
Source: Front Immunol. 2025 Nov 27;16:1676937. doi: 10.3389/fimmu.2025.1676937 (PMC12695756; doi:10.3389/fimmu.2025.1676937)

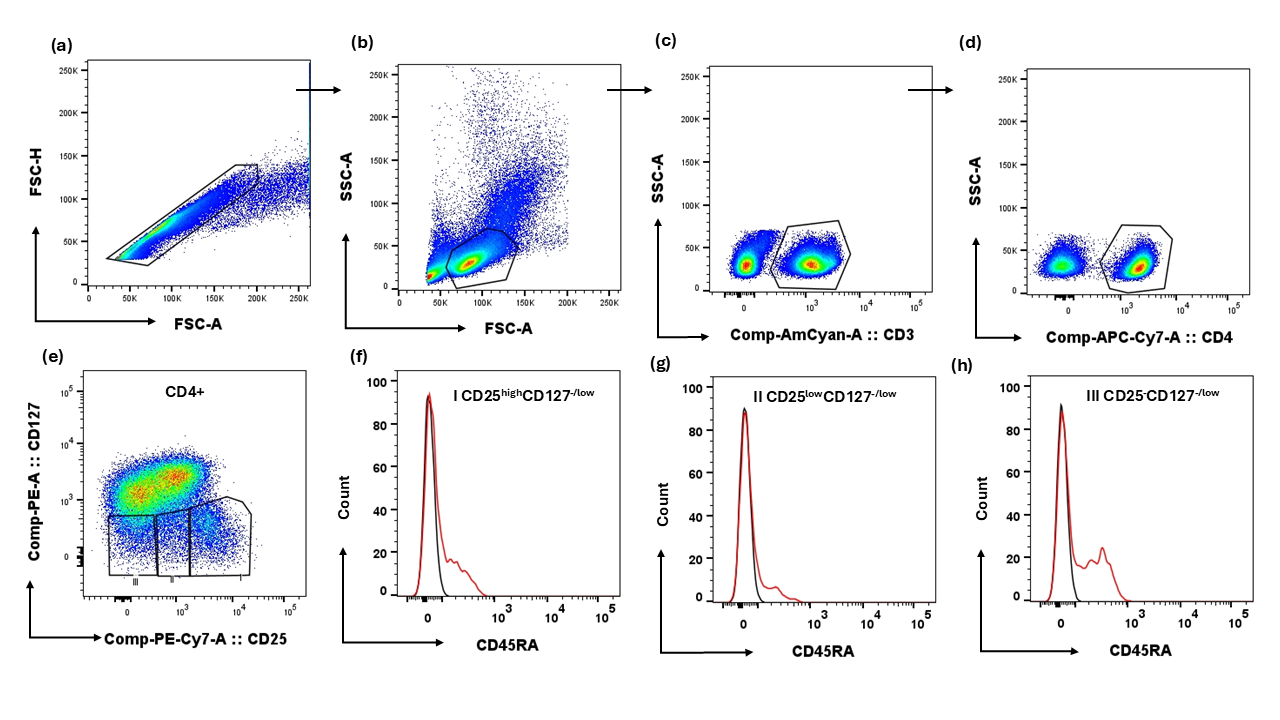

Supplement: Supplementary Figure 1 — Gating strategies for identification of different subsets of Treg and CD45RA staining. Fresh peripheral blood was stained ex vivo for CD3, CD4, CD127, CD25 and CD45 RA for multiparametric analysis through FACS. CD3+ and CD4+ cells were gated from the single cell population of T lymphocytes (a–d). CD3+CD4+ population was gated with low expression of CD127 (CD3+CD4+CD127-/low) and variable expression of CD25, CD25-, CD25low and CD25high(e). Percentage of CD45 RA positive cells in the CD25high, CD25low, and CD25- cell populations are shown as histograms (f–h). Fluorescence minus one (FMO) staining was used both for gating CD127 and CD45 RA. Gating strategy is illustrated using a representative healthy control sample. [file Image1.tif]

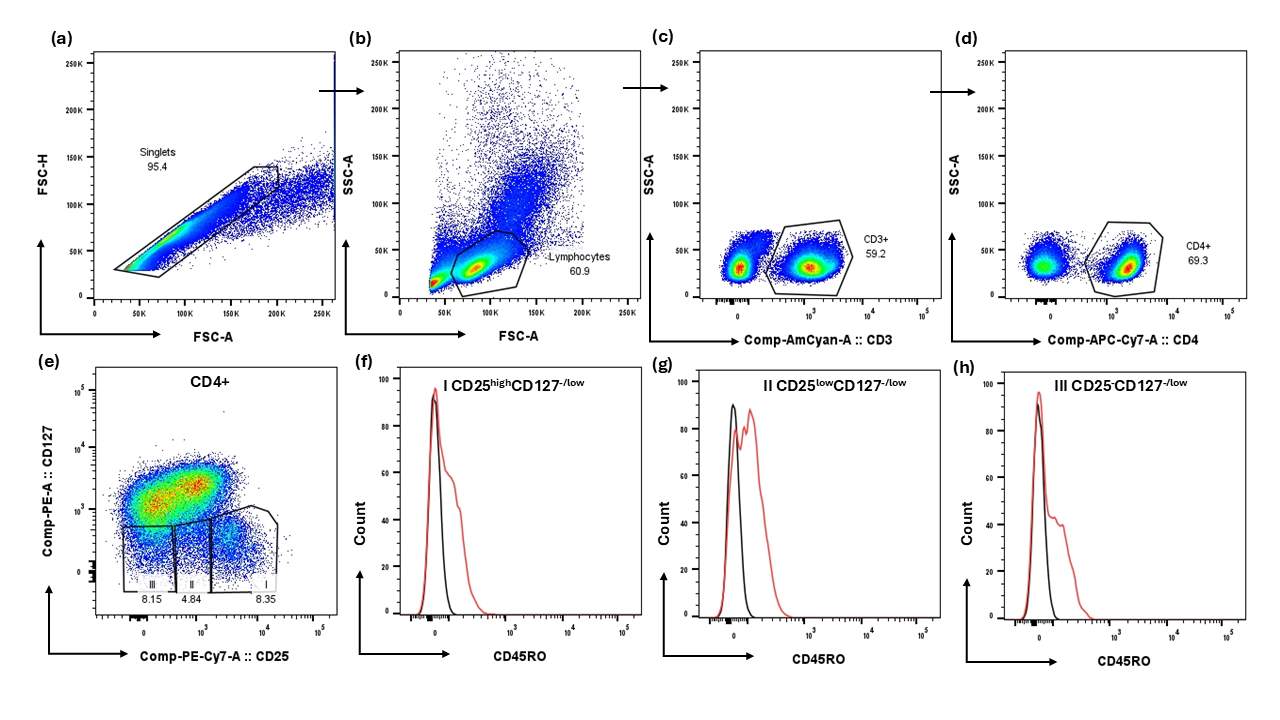

Supplement: Supplementary Figure 2 — Gating strategies for identification of different subsets of Treg and CD45RO staining. Fresh peripheral blood was stained ex vivo for CD3, CD4, CD127, CD25 and CD45 RO for multiparametric analysis through FACS. CD3+and CD4+ cells were gated from the single cell population of T lymphocytes (a–d). CD3+CD4+ population was gated with low expression of CD127 (CD3+CD4+CD127-/low) and variable expression of CD25, CD25-, CD25low and CD25high(e). Percentage of CD45 RO positive cells in the CD25high, CD25low, and CD25- cell populations are shown as histograms (f–h). Fluorescence minus one (FMO) staining was used both for gating CD127 and CD45 RO. Gating strategy is illustrated using a representative healthy control sample. [file Image2.tif]

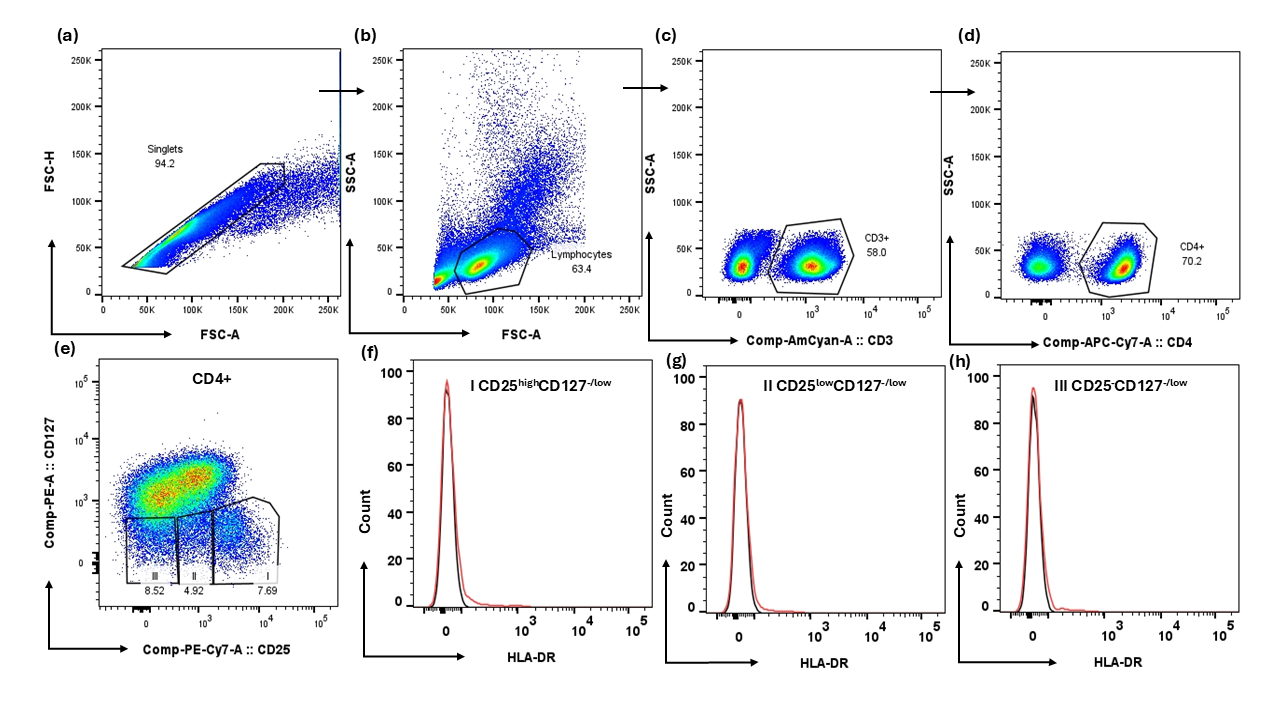

Supplement: Supplementary Figure 3 — Gating strategies for identification of different subsets of Treg and HLA-DR staining. Fresh peripheral blood was stained ex vivo for CD3, CD4, CD127, CD25 and HLA-DR for multiparametric analysis through FACS. CD3+and CD4+ cells were gated from the single cell population of T lymphocytes (a–d). CD3+CD4+ population was gated with low expression of CD127 (CD3+CD4+CD127-/low) and variable expression of CD25, CD25-, CD25low and CD25high(e). Percentage of CD95 positive cells in the CD25high, CD25low, and CD25- cell populations are shown as histograms (f–h). Fluorescence minus one (FMO) staining was used both for gating CD127and HLA-DR. Gating strategy is illustrated using a representative healthy control sample. [file Image3.tif]

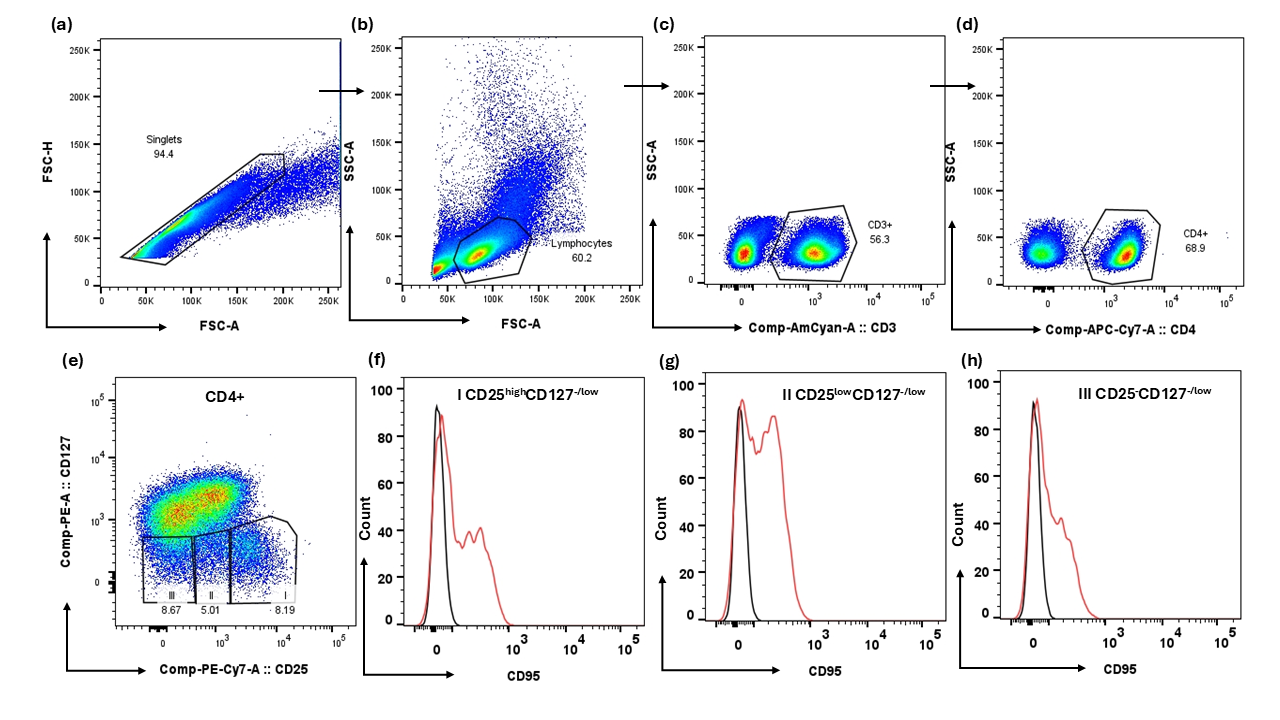

Supplement: Supplementary Figure 4 — Gating strategies for identification of different subsets of Treg and CD95 staining. Fresh peripheral blood was stained ex vivo for CD3, CD4, CD127, CD25 and CD95 for multiparametric analysis through FACS. CD3+and CD4+ cells were gated from the single cell population of T lymphocytes (a–d). CD3+CD4+ population was gated with low expression of CD127 (CD3+CD4+CD127-/low) and variably expression CD25, CD25-, CD25low and CD25high(e). Percentage of HLA-DR positive cells in the CD25high, CD25low, and CD25- cell populations are shown as histograms (f–h). Fluorescence minus one (FMO) staining was used both for gating CD127and CD95. Gating strategy is illustrated using a representative healthy control sample. [file Image4.tif]

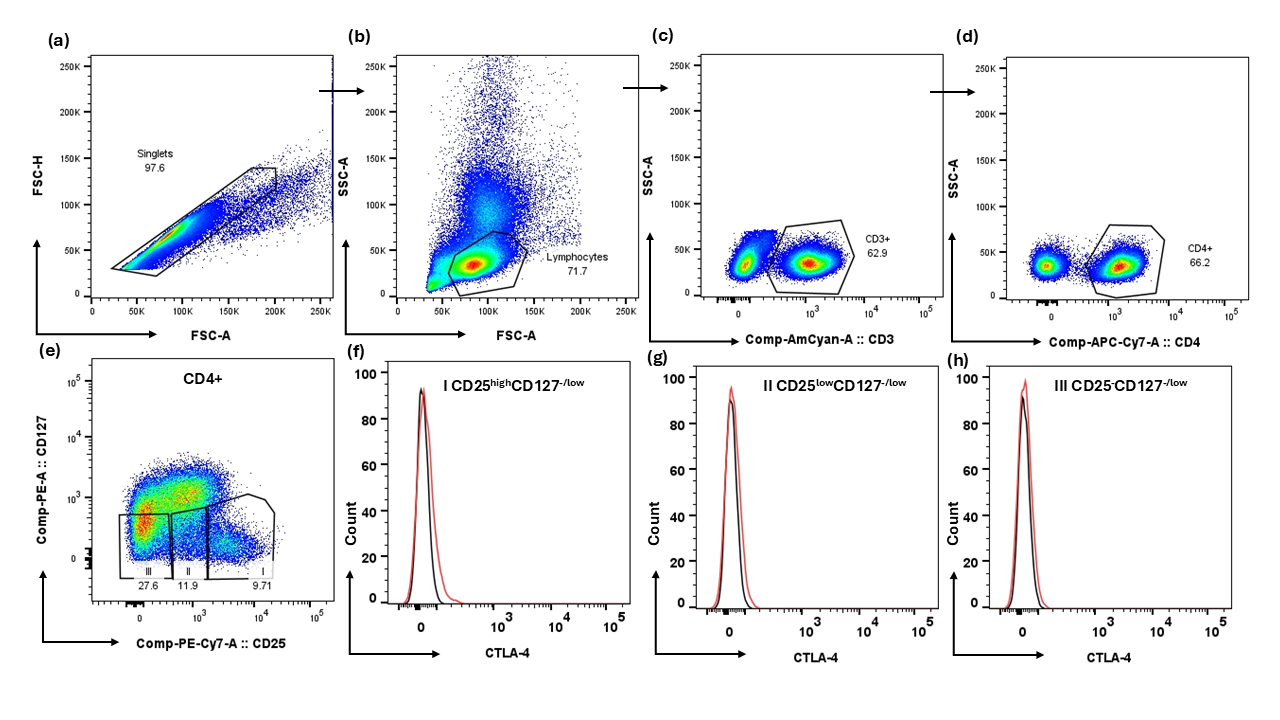

Supplement: Supplementary Figure 5 — Gating strategies for identification of different subsets of Treg and CTLA-4 staining. Fresh PBMCs were stained ex vivo for CD3, CD4, CD127 and CD25 followed by intracellular staining for CTLA-4 T cells. CD3+and CD4+ cells were gated from the single cell population of T lymphocytes (a–d). CD3+CD4+ population was gated with low expression of CD127 (CD3+CD4+CD127-/low) and variable expression of CD25, CD25-, CD25low and CD25high expression (e). Percentage CTLA-4 positive cells in the CD25high, CD25low, and CD25- cell populations are shown as histograms (f–h). Fluorescence minus one (FMO) staining was used both for gating CD127 and CTLA-4. Gating strategy is illustrated using a representative healthy control sample. [file Image5.tif]
